# Needling Interventions for Sciatica: Choosing Methods Based on Neuropathic Pain Mechanisms—A Scoping Review

**DOI:** 10.3390/jcm10102189

**Published:** 2021-05-19

**Authors:** Thomas Perreault, César Fernández-de-las-Peñas, Mike Cummings, Barry C. Gendron

**Affiliations:** 1 Northern New England Spine Center, Department of Physical Therapy, Wentworth Douglass Hospital, Dover, NH 03820, USA; thomas.perreault@wdhospital.org; 2 Department of Physical Therapy, Occupational Therapy, Rehabilitation and Physical Medicine, Universidad Rey Juan Carlos, 28922 Alcorcón, Madrid, Spain; 3 Cátedra Institucional en Docencia, Clínica e Investigación en Fisioterapia: Terapia Manual, Punción Seca y Ejercicio Terapéutico, Universidad Rey Juan Carlos, 28922 Alcorcón, Madrid, Spain; 4 British Medical Acupuncture Society, London WC1N 3HR, UK; mike.cummings@btinternet.com; 5 Northern New England Spine Center, Department of Physical Medicine and Rehabilitation, Musculoskeletal Health and Rehabilitation, Wentworth Douglass Hospital, Dover, NH 03820, USA; barry.gendron@wdhospital.org

**Keywords:** sciatica, neuropathic pain, dry needling, acupuncture, mechanisms

## Abstract

Sciatica is a condition often accompanied by neuropathic pain (NP). Acupuncture and dry needling are common treatments for pain, and the current literature supports acupuncture as an effective treatment for sciatica. However, it is unknown if the mechanisms of NP are considered in the delivery of needling interventions for sciatica. Our objective was to assess the efficacy and the effectiveness of needling therapies, to identify common needling practices and to investigate if NP mechanisms are considered in the treatment of sciatica. A scoping review of the literature on needling interventions for sciatica and a review of the literature on mechanisms related to NP and needling interventions were performed. Electronic literature searches were conducted on PubMed, MEDLINE, CINAHL and Cochrane Database of Systematic Reviews from inception to August, 2020 to identify relevant papers. Reference lists of included papers were also manually screened and a related-articles search through PubMed was performed on all included articles. Mapping of the results included description of included studies, summary of results, and identification of gaps in the existing literature. Ten articles were included. All studies used acupuncture for the treatment of sciatica, no studies on dry needling were identified. Current evidence supports the efficacy and effectiveness of acupuncture for sciatica, however, no studies considered underlying NP mechanisms in the acupuncture approach for sciatica and the rationale for using acupuncture was inconsistent among trials. This review reveals that neuropathic pain mechanisms are not routinely considered in needling approaches for patients with sciatica. Studies showed acupuncture to be an effective treatment for sciatic pain, however, further research is warranted to explore if needling interventions for sciatica and NP would be more effective if NP mechanisms are considered.

## 1. Introduction

Sciatica is a term commonly used to describe radiating pain in the leg [1]. Historically, the term ‘sciatica’ was used to describe pains or ‘ischias’ felt around the hip or thigh [2]. Sciatica is closely associated with pain and/or sensory symptoms along a dermatome or the innervated region of an affected spinal root or roots [3,4]. Prevalence estimates for sciatica are heterogeneous, with an annual prevalence between 2.2% and 34% [5]. A leading cause of sciatica is distortion of a lumbar nerve root due to disc rupture and the literature affirms that the terms sciatica and radiculopathy are often used interchangeably and inconsistently [6]. According to an epidemiological study, sciatica is highly prevalent in patients with nonlocalized low back pain, i.e., pain occurring at another anatomical site in addition to the low back [7]. Porchet et al. reported that over 90% of patients with sciatica had MRI confirmed disc bulge, disc protrusion, extrusion or sequestration [8]. The L4-L5 and L5-S1 discs were pathologic in 36% and 53% of these patients, respectively [8]. Along with radicular pain, sensory loss [9], myotome and reflex deficits are common findings on clinical examination in patients with MRI confirmed discogenic sciatica [10]. According to the International Association for the Study of Pain (IASP), painful radiculopathy is an entity of neuropathic pain (NP) as the diagnosis requires pain or sensory symptoms along affected dermatomes or nerve roots [11]. Importantly, a recent study found that NP was present in 48% to 74% of patients with sciatica seeking treatment in primary care [12].

It has been proposed that treatment strategies for NP should be tailored and based on addressing potential underlying mechanisms [9]. In the literature, NP is often studied using chronic constriction injury (CCI) of the sciatic nerve in animal models. Interestingly, CCI models imitate the compression-induced nerve injury seen in sciatica and related NP conditions in humans [13]. Since sciatica can involve nerve root compression and symptoms consistent with NP, a mechanism-based-approach is called for to provide optimal management strategies. However, the features of NP are unique, vary across NP conditions [9] and can be difficult to manage. Mechanistically, spinal disinhibition resulting from nerve injury leads to decreased control over nociceptive [14] and non-nociceptive inputs [15]. CCI induces pathological changes in the dorsal horn that enhance excitatory synaptic transmission while impairing the inhibitory capacity of dorsal horn neurons (DHN) in lamina Ⅱ [16].

Mechanistic studies have investigated acupuncture effects using CCI and similar models of NP [17,18,19], providing evidence that acupuncture alleviates mechanical allodynia [20] and dose-dependently gives rise to antinociceptive effects [21,22,23]. For patients with sciatica, acupuncture is one of the most effective treatments for pain reduction [24]. Like acupuncture, dry needling is a unique method of neural stimuli that evokes sensory nerve impulses. These impulses conduct to DHN by way of the needle interaction with nearby nerves [25] and connective tissues [26] leading to activation of endogenous pain mechanisms [27]. Although dry needling and acupuncture differ in theoretical constructs, both interventions use a needle as a mechanical-stimuli to primarily treat pain [28]. Furthermore, points of needle insertion overlap between the procedures in terms of anatomical relevance and clinical uses for the treatment of pain [29,30]. This review will discuss acupuncture and dry needling together as they relate to the treatment of sciatic pain. Segmental inhibitory mechanisms in the dorsal horn explain the analgesic effects of acupuncture and dry needling [29,31,32,33]. However, in animal models of nerve injury the ability for Aδ and C-fibers to activate inhibitory interneurons in the dorsal horn is attenuated [34], pain modulation from endogenous opioids is impaired [35] and GABA and glycine receptor function is altered [36]. Therefore, impaired segmental inhibition deserves consideration during the application of needling interventions for patients with sciatica and NP.

With that aim, a scoping review of the literature was conducted to identify if NP mechanisms are considered in the treatment rationale and/or application of acupuncture or dry needling interventions for the treatment of sciatica-related pain. This scoping review was conducted to address the following questions: (1) does the available evidence support the efficacy and effectiveness of acupuncture or dry needling for sciatica-related pain? (2) what are the most common needling parameters and locations used for treating sciatica? (3) are neuropathic pain mechanisms considered in the rationale or treatment approaches for needling interventions? In addition, a review of literature on mechanisms related to NP and needling interventions was performed to support clinical considerations in the application of needling for sciatica related NP.

## 2. Methods

### 2.1. Scoping Review

This scoping review followed the methodological framework for scoping reviews outlined by the Preferred Reporting Items for Systematic Reviews and Meta-Analyses Extension for Scoping Reviews (PRISMA-ScR) [37].

### 2.2. Search Strategy

Electronic literature searches were conducted using the following databases from inception to August 2020; PubMed, MEDLINE, CINAHL and Cochrane Database of Systematic Reviews. Reference lists of included papers were also manually screened. An experienced health science librarian was consulted for guidance on search strategies. In the search formulas the following terms were merged using Boolean operators; “Dry Needling”, “Acupuncture”, “Neuropathic Pain”, “Sciatica”, “Radiculopathy”, “Radiculopathic”. See Table 1 for electronic database search formulas. Consistent with our intent to also review the literature related to mechanisms of needling interventions and NP, electronic literature searches were conducted using the following databases from inception up to August 2020; Google Scholar, PubMed, MEDLINE and CINAHL. The following terms were also merged into the search formulas using Boolean operators; “Neuropathic Pain”, “Dry Needling”, “Acupuncture”, “Mechanisms”, “Adenosine”, “Noradrenaline”, “Segmental”, “Supraspinal”. In addition, reference lists of selected articles were manually screened to identify additional studies. Further, a related-articles search through PubMed was performed on all included articles. 

### 2.3. Inclusion/Exclusion Criteria

We included (1) systematic reviews, meta-analyses, and randomized controlled trials published in English evaluating the efficacy or effectiveness of dry needling or acupuncture for the treatment of sciatica; (2) review studies with other spinal conditions were accepted only if results on sciatica or lumbar radiculopathy were clearly displayed. The following studies on mechanisms of needling interventions and NP were considered: systematic reviews and meta-analyses, randomized controlled trials, narrative reviews, experimental in vivo animal studies, clinical studies, laboratory studies, modeling and simulation studies. A single author (TP) reviewed the identified publications by assessing the title and abstract. Relevant papers were included or excluded using the criteria listed in Table 2. A second author (BG) was consulted during the process of article inclusion if questions arose as to the relevance of particular studies. No discrepancy occurred between both authors with regards to study selection and there was no need for a third author to be consulted. Studies were eliminated based on the exclusion criteria in Table 2.

### 2.4. Data Mapping

After thorough review of all studies we organized the topics accordingly; (1) synthesis of the data on the effectiveness and efficacy of dry needling and/or acupuncture for sciatica, that is, we displayed pain reduction from the needling interventions within the context of routine care or under ideal conditions, respectively, (2) data for patient inclusion, treatment rationale and clinical applications of dry needling or acupuncture, (3) summary of needling interventions in the context of pain related to sciatica and NP considerations, (4) provided a mechanisms-based approach for the use of dry needling or acupuncture for sciatica. Because some of the selected studies were systematic reviews and/or meta-analyses, the discussion of data related to treatment protocols and acupuncture point selection will be presented as the original authors have summarized in their studies. For example, the majority of articles that are included in the systematic reviews were written in Chinese or another language, and the full text could not be read. Only the articles that fit our original search criteria and could be read and accessed as full text were individually discussed, regardless of whether they were also part of one of the included systematic reviews/meta-analyses.

## 3. Results

**Figure 1:**
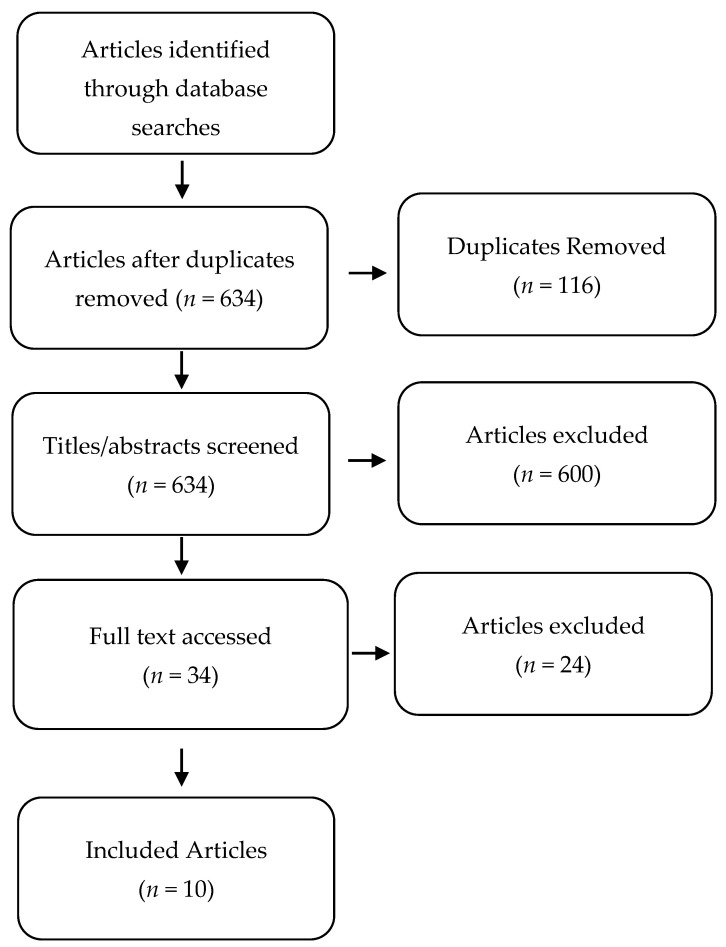
Flow diagram outlining selection of studies based on PRISMA guidelines.

The initial database searches identified 750 potential articles. After removing duplicates (*n* = 116) 634 articles remained for screening. Six hundred articles were excluded based on review of the titles and abstracts, leaving 34 articles for full text review. Following further exclusion of 24 articles, 10 articles were included for discussion, and consisted of systematic review and meta-analysis studies (*n* = 4), systematic reviews (*n* = 2), randomized controlled trials (*n* = 3) and a randomized controlled pilot study (*n* = 1). Figure 1 shows the flow diagram based on PRISMA guidelines.

Using our inclusion criteria, no studies on dry needling were identified. All the included studies (*n* = 10) used acupuncture for treatment of sciatica and are outlined in Table 3 and Table 4. The inclusion criteria for patients with sciatica varied across the studies. Five studies included participants with sciatica symptoms and clinical findings that correlated with MRI or CT confirmation of lumbar disc herniation [22,38,39,40,41]. Three studies included patients with a clinical diagnosis of sciatica based on nerve root pain and referred pain [24,42,43]. Lastly, in two systematic review and meta-analysis studies the conformity of inclusion criteria was limited in the included trials. Ji et al. included studies with participants who had either subjective signs of sciatica, positive clinical examination tests or both [44]. Qin et al. included patients with sciatica of the nerve roots along with lumbar disc herniation or with sciatica of the nerve trunk without lumbar disc herniation [45]. Liu et al. used the most specific criteria by selecting patients with radicular pain in L4, L5, or S1 dermatome, physical examination results consistent with L4, L5, or S1 spinal nerve root involvement and MRI demonstrating a unilateral disc herniation impinging on the L4, L5, or S1 nerve roots [22].

### 3.1. Effects on Pain Intensity

According to the most updated review, Huang et al. reported that for patients with sciatica, acupuncture showed favorable reduction in pain intensity at immediate weeks) follow up (weighted mean difference (WMD) −11.94, 95% confidence interval (CI) −13.22 to −10.67, I^2^ 0%). In addition, significant reduction in pain was demonstrated at short-term (<3 months) (WMD −8.90, CI −17.28 to −0.52, I^2^ 84.4%), med-term (3–6 months) (WMD −17.80, CI −19.51 to −16.60, I^2^ 93.0%), and long-term (>6 months) (WMD −17.60, CI −19.23 to −15.97, I^2^ 0%) follow ups (moderate quality evidence) [40]. Included studies for sciatica all favored acupuncture for pain compared to physical therapy, electrotherapy, and sham acupuncture. Pooled data from 3 trials on sciatica was used for subgroup analysis with 2 trials using a 0–100 mm VAS scale and 1 using an 11-point numerical rating scale. 

### 3.2. Efficacy

The randomized controlled trial by Huang et al. compared the effects of a 4-week course of real vs. sham acupuncture in patients with sciatica. They reported a significant between-groups difference for leg pain on the 0–100 mm VAS at week 4 (–11.25 mm, 95%CI −21.06 to −1.44, *p* = 0.026), favoring real acupuncture. Sham acupuncture involved blunt tipped needles without insertion onto acupoints. In addition, a mean change in leg pain over 4 weeks from baseline was −22.22 mm (95%CI −26.30 to −8.14) in the acupuncture group and −14.94 mm (95%CI −20.21 to −9.66) in the sham acupuncture group, with a between-group difference of −7.28 mm (95%CI −13.76 to −0.80, *p* = 0.029) also favoring real acupuncture [41]. These results are consistent with the effects of acupuncture compared to sham across several common pain conditions [46].

A network meta-analysis by Lewis et al. compared 21 treatment strategies for sciatica. Acupuncture was favored for reduction in pain intensity compared with inactive controls (WMD: −25, 95%CI −41.75 to −8.25) and had the second highest probability of being superior for reducing pain compared with other interventions [24]. However, of the 122 studies included, only a single study on acupuncture was analyzed to determine results on pain intensity and the pain scale used was not reported [47]. In an earlier systematic review, real acupuncture demonstrated significant improvement in pain intensity compared to sham acupuncture (WMD −25.00, 95%CI −41.19 to −8.81) but the pain scale used in the included trial was not reported [42]. In contrast, Luijsterburg et al. concluded in the earliest included review that there was insufficient evidence supporting electroacupuncture for pain in patients with lumbar radicular syndrome based on limited data reported in a single study [43].

### 3.3. Effectiveness

In the meta-analysis by Ji et al. acupuncture was more effective than conventional medication for individuals with sciatica for pain intensity (mean difference (MD) −1.25; 95%CI −1.63 to −0.86, *n* = 3 trials) and for pain threshold (MD: 1.08; 95%CI 0.98 to 1.17, *n* = 3 trials using 0–100 mm VAS) [44]. However, most randomized trials had low methodological quality and high risk of bias. Additionally, treatment regimens with acupuncture were highly variable between trials. According to the meta-analysis by Qin et al., compared to NSAIDs, acupuncture was more effective for decreasing leg pain (MD −1.23, 95%CI −1.87 to 0.60, 𝐼^2^ 0%, *n* = 3 trials using 0–100 mm VAS) [45].

Zhang et al. reported that electroacupuncture (EA) was superior for reducing mean leg pain intensity compared with electrotherapy. Specifically, the mean change in pain intensity on the 11-point NRS from baseline to week 4 was 2.30 (1.86–2.75) in EA group and 1.06 (0.62–1.51) in electrotherapy group [38]. Importantly, at week 28 follow-up only the EA group showed significantly decreased leg pain compared to the baseline, implying that the effect of EA but not electrotherapy lasted for 28 weeks. Jeong et al. reported that acupuncture led to reductions in pain of 8.13 and 12.28 mm on the VAS at 4 and 6 weeks from baseline, respectively, in 70 patients with radicular symptoms consistent with lumbar disc herniation [39].

In a recent randomized controlled study on 30 subjects with chronic sciatica, high dose acupuncture (18 acupoints) was compared with low dose acupuncture (6 acupoints) over a 4-week period. After eight sessions in each group, leg pain at week 4 showed significant within-group reductions on the 0–100 mm VAS (high dose from 6.29 ± 1.76 to 4.00 ± 1.95, *p* < 0.005) and (low dose from 5.90 ± 1.74 to 3.94 ± 2.51, *p* < 0.05) without significant between-groups differences [22]. While the number of needles inserted is one of the only dosage parameters attributed to better outcomes in some chronic pain conditions [48] this has not been demonstrated in patients with sciatica or NP.

### 3.4. Common Needling Practices for Sciatica

None of the included studies reported consideration of or accommodations for NP mechanisms in the acupuncture approach for sciatica. Additionally, the rationale for using acupuncture was inconsistent among studies. Two systematic reviews reported that all included trials used Traditional Chinese Medicine (TCM) theory [44,45], two selected points based on expert consensus and a previous study protocol [38,41], and one study used acupuncturist experience in addition to TCM theory [22]. In four studies the treatment rationale for use of acupuncture was either not explicitly stated [39,49] or could not be determined due to language barriers [24,42,43]. Interestingly, one recent trial applied a Western medicine approach by choosing acupuncture points for sciatica that were distributed along the L4, L5 and S1 dermatomes in addition to rationale based on TCM theory [22]. In addition, one study placed needles bilaterally at the segmental level of lumbar disc herniation in combination with BL25 [38].

**Figure 2:**
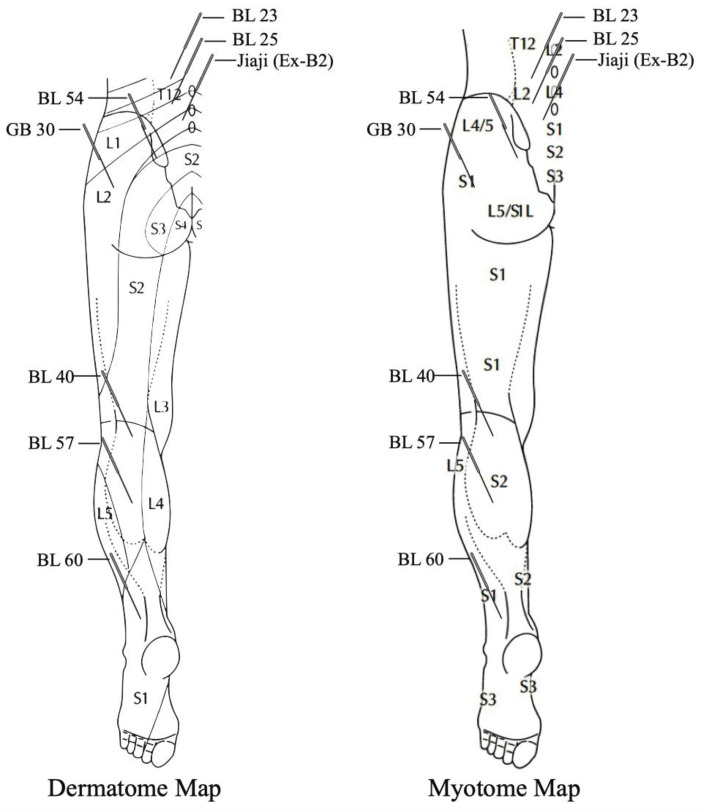
Schematic illustration of acupuncture points most commonly used across studies in their anatomic dermatome and myotome region. BL: bladder; Jiaji: huatuojiaji; GB: gallbladder.

Regarding type of needle stimulation, EA was most frequently used in the included trials in the systematic reviews and meta-analysis studies followed by acupuncture. Within the selected randomized controlled trials, three used acupuncture exclusively [22,39,41] while one reported manual manipulation followed by electric needle stimulation [38]. Most reported the elicitation of de qi (needle sensation) and needle manipulations varied across studies between lift-thrust, twirling or a combination of both [22,39,40]. Needle retention time ranged from 5–45 min between included studies in the reviews and between 15–30 min in the randomized controlled trials. Frequency, duration and number of needling sessions were inconsistent across trials, and the number of treatments ranged between four to sixteen. Collectively, the included studies demonstrate the following; (1) multiple needles are inserted intentionally or coincidentally along the sciatic nerve distribution and associated dermatomes and/or myotomes, (2) needles are manipulated either manually or electrically, (3) needles are retained for a predetermined duration with or without intermittent manipulation, (4) multiple sessions are provided within a 2–4 week period. At present, no dosage guidelines for acupuncture or dry needling exists for the treatment of sciatica or NP. However, consistent with a recent expert consensus on acupuncture for sciatica [50], the included studies used common acupuncture points for sciatica along the gall bladder (GB) and bladder (BL) meridians that are depicted in Figure 2.

## 4. Discussion

This scoping review highlights the evidence for acupuncture as an effective treatment for sciatic pain, yet no studies on dry needling were located. We identified that NP mechanisms are not considered in the acupuncture approaches for sciatica. In addition, needling locations and specific treatment parameters are selected based on TCM theory and expert consensus. The GB and BL meridians are the key meridians in acupuncture for treating sciatica and are distributed along the dermatomes and myotomes related to the sciatic nerve [4]. There is clearly some neuroanatomical significance to the meridians and acupuncture points most commonly chosen for sciatica [51]. Needling beyond and not at the site of compression is common in practice as most points being needled for sciatica are distal or proximal to sites of potential nerve compression. This approach has shown to inhibit nociception in CCI models of neuropathic pain lending support to the common acupuncture practices for sciatica [13]. For sciatica, placement of needles along the dermatomes, myotomes and spinal segments related to the sciatic nerve seems to be effective [52].

In the following sections, we discuss the results of the narrative review about mechanisms on NP for the management of sciatica.

### 4.1. Disinhibition: Considerations for Needling Interventions

Evidence supports that needle stimuli triggers release of endogenous opioids [21,53] from DHN [54] that are evoked during needle manipulation [55,56,57]. Studies show opioid peptides are released only in spinal segments that receive innervations from the stimulated area, ipsilateral to the mechanical stimulus [27,58]. Consequently, the needle evoked opioid release produces strong inhibitory effects at the spinal cord level [59,60]. However, the neural drive along injured afferent fibers is reduced limiting the ability for noxious stimulation to activate target receptors in the dorsal horn [61]. According to Chen et al. [61], μ-opioid receptor (MOR) inhibition of neuropeptide release from afferent terminals in the dorsal horn is impaired following CCI. Importantly, downregulation of MOR expression occurs in the spinal segment of injured fibers and in neighboring rostro-caudal segments ipsilateral to the injury [62]. Thus, inhibition of DHN from noxious stimulation induced release of endogenous opioids is impaired in the dorsal horn following nerve injury [63]. Given this, clinicians should carefully consider using painful needle stimuli at the segmental level(s) of nerve injury or compression to elicit opioid related effects in patients with sciatica and NP.

Primary afferent input is also modulated by an inter-neuronal pathway dependent on gamma-aminobutyric acid (GABA), glycine, and their respective receptors [64,65]. However, studies show that disruption of potassium-chloride co transporter 2 (KCC2) in DHN alters the function of GABA and glycine leading to disinhibition [36,66]. Experimental manipulation of chloride (CL−) channels replicates the same sciatic nerve injury associated sensory miscoding and tactile allodynia [64,67]. However, restoring CL- mediated inhibition of DHN by application of a KCC2 activator after nerve injury reversed signs of tactile allodynia in an animal model [15]. Prior evidence suggests that after nerve injury, tactile allodynia results from Aβ fibers sprouting into nociceptive pathways in the superficial lamina [68,69,70]. Yet, evidence for nerve injury-induced Aβ fiber sprouting is lacking and suggests other mechanisms [71]. A novel study demonstrated that glycinergic inhibition of protein kinase c gamma (PKCÝ) neurons is impaired at the spinal level of nerve injury leading to tactile allodynia via Aβ fiber activation of PKCÝ neurons [72]. These studies showcase the problem of disinhibition and tactile allodynia that occur in NP. Using needle approaches that specifically target Aβ fiber activation, such as innocuous EA, may fail to produce analgesic effects if the segmental area of pain is directly targeted [31]

### 4.2. Needle Manipulation, Mast Cells and Mediators

Evidence supports that collagen fibers transmit mechanical signals to afferent fibers during needle stimuli [73,74,75,76] and that the analgesia produced occurs via connective tissue winding [77,78]. In contrast, Chang et al. found that needle manipulation still induced antinociceptive effects despite destruction of local collagen fibers [25]. Chen et al. demonstrated that some afferent fibers are responsive to local needling by mechanical activation of transient receptor potential vanilloid 1 (TRPV1) receptors evoking excitatory responses that propagate to the dorsal root ganglion (DRG), DHN and to the somatosensory cortex [79]. Thus, needle stimuli may directly activate primary afferents by distortion of nerve terminals near the needle and not just through connective tissue mechanisms [80]. A recent study reported that mast cell (MC) degranulation was essential to evoke nerve discharges in the lumbar dorsal roots during needle manipulation. That is, MC degranulation results in release of MC mediators, such as ATP and histamine, that trigger nerve discharges [26]. In mechanistic studies, MC are reported to be essential to the therapeutic effects of needling [74,81,82]. The presence of mechanosensitive channels on MC allow a variety of stimuli to trigger degranulation [83,84,85]. MC activation can be triggered by elevated interstitial fluid pressure and fluid shear stress nearby an inserted needle [86,87]. Dimitrov et al. reported in rats that MC degranulation occurred along the needle tract following insertion without manipulation [88]. Importantly, manipulation applied to needles after insertion will alter tissue stiffness and sustain MC recruitment and degranulation near the needled area [89]. Earlier studies on animal [17] and human models [90] found that needling increases local ATP and adenosine concentrations. More recent studies support that MCs, fibroblasts and afferent fibers are the primary sources of needle induced ATP release [85,91,92,93]. Interestingly, when MCs are exposed to extracellular ATP it will bind to P2_Y13_ and P2_x7_ receptors on the same or nearby MCs to further liberate intracellular ATP stores [94]. This creates a renewable purinergic signal along connective tissues and afferent pathways. In theory, needle retention interrupted by bouts of manipulation may sustain MC degranulation and subsequent release of ATP.

### 4.3. Adenosine Modulates Neuropathic Pain

Studies show extracellular ATP is rapidly broken down into adenosine, which has antinociceptive effects locally and at the spinal level by activating the adenosine A1 receptor (A1R) [17,90,95,96]. Some A1Rs are located on primary afferent terminals, but most are confined to interneurons in lamina Ⅱ [97] and when activated by adenosine will hyperpolarize interneurons receiving Aδ and C-fiber input [98,99]. Due to their position, A1Rs have been shown to modulate Aβ fiber activity and reduce tactile allodynia under conditions of NP [20]. Adenosine also acts on A1Rs of afferent terminals to inhibit substance P (SP), calcitonin gene related peptide (CGRP) and excitatory neurotransmitter release [100] making it useful for NP by counteracting increased neuropeptide expression that occurs following nerve injury [13]. Evidenced demonstrates that ectonucleotidases reside within the epineurium of peripheral nerves, the membrane of DRG neurons and their axon terminals in lamina Ⅱ, which hydrolyze nucleotides into adenosine [95,96,101]. Following nerve injury, the expression of these enzymes is downregulated [102] in the laminar region containing axon terminals from the injured afferents [95]. Consequently, this impairs adenosine analgesia in the injured spinal segment. Theoretically, producing a build-up of adenosine via needling along non-injured rostro-caudal segments could overwhelm adenosine transporters and enzymes [96] while also aiding adenosine analgesia at injured segments.

### 4.4. Intersegmental Approach

There is some evidence of orderly arrangement in the pathway and synaptic termination of afferent fibers connecting to the dorsal horn. Shehab et al. demonstrated that Aδ, Aβ and C fibers in the L4–L5 dorsal roots project to similar rostro-caudal locations and terminate in a distinct laminar distribution within the L3–L5 spinal segments [103]. However, individual primary afferents also ramify at the dorsal roots spreading several segments along a rostro caudal axis, after root branching, prior to entering specific lamina at the dorsal horn. A recent study reported afferent fiber arborization can occur up to six spinal segments apart before they integrate into the superficial dorsal horn [104]. Within the superficial lamina individual nerve fibers contain many synaptic boutons and give off ascending collateral branches that synapse with interneurons of neighboring segments along with afferent terminals from the segment of origin [105,106]. Thus, the circuitry within the dorsal horn functions inter-segmentally. In fact, interneurons in lamina Ⅱ receive monosynaptic Aδ and C fiber inputs from arborizations of up to four different dorsal roots [107] and neurons in lamina Ⅰ receive monosynaptic innervations from up to six dorsal roots [108]. That is, many afferent fibers arrive at the dorsal horn at an entirely different segment before terminating at a given level. Pinto et al. found that the strongest Aδ and C fiber inputs to neurons in lamina Ⅰ were from arborizations coming from the root of the caudal segment, followed by the root projecting to the spinal segment containing that neuron [108]. Fernandes et al. reported that C fiber inhibition is strongest at a given segment when afferents of a caudal segment that project to it are activated via noxious stimulation [109]. Interestingly, needling along an area with sensory projection spanning within the rostro-caudal extent of 2–3 spinal segments of an injured spinal nerve has proved to relieve mechanical hypersensitivity in animal models of NP [18]. 

**Figure 3:**
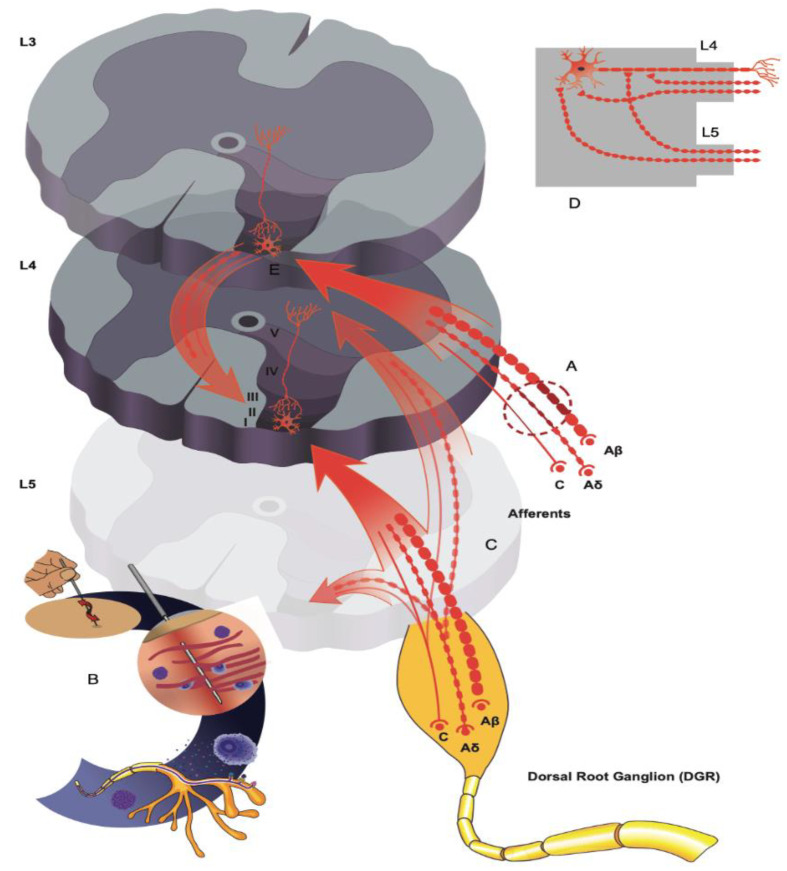
Schematic illustration of neuropathic pain mechanisms and intersegmental needling. (**A**) Nerve injury downregulates MOR and KCC2 expression in the dorsal horn weakening segmental inhibition. (**B**) Needle insertion with retention or manipulation activates mechanosensitive channels on afferent fibers, MC and fibroblasts promoting release of ATP. ATP is broken down into adenosine to provide antinociceptive effects locally and at the spinal level by activating A1Rs on primary afferent terminals and interneurons in lamina Ⅱ. (**C**) Needle stimuli aimed at rostro-caudal segments away from the primary segment of nerve injury will activate Aδ and C fiber arborizations of neighboring roots that synapse with interneurons in lamina Ⅰ-Ⅱ at the segment of injury (**D**) Needle induced increases of GABA, NA and adenosine potentiate presynaptic and postsynaptic analgesic effects through volume transmission. (**E**) Needle stimuli will inhibit microglial activation leading to downregulation of the BDNF and TrkB pathway, increasing KCC2 expression in the lumbar dorsal horn at the segment of nerve injury, and restoring chloride regulation in dorsal horn neurons. MOR, μ-opioid receptor; KCC2, potassium-chloride co transporter 2; MC, mast cells; ATP, adenosine triphosphate; A1Rs, A1 receptors; Aδ, A delta; GABA, gamma-aminobutyric acid; NA, noradrenaline; BDNF, brain-derived neurotrophic factor; TrkB, tyrosine kinase receptor B.

Activation of interneurons in lamina Ⅰ–Ⅱ through multiple afferent inputs triggers the release of endogenous GABA resulting in presynaptic inhibition at primary afferent terminals by way of GABA volume transmission [110] and binding onto GABA_B_ receptors [111,112]. GABA_B_ receptors on Aδ and C fiber terminals have proven to attenuate nociceptive transmission into the dorsal horn by suppressing glutamate and SP release and through hyperpolarization of DHN [113,114]. Importantly, GABA_B_ receptors are not susceptible to the CL− dysregulation that occurs following nerve injury. A recent study demonstrated that electroacupuncture applied within a particular lumbosacral dermatome, synchronously activates DHN that are interconnected in a rostro-caudal manner across multiple spinal segments [115]. Evidence suggests that presynaptic inhibition is enhanced by increasing the synchronization between pairs of evoked DHN, allowing better modulation of arriving afferent inputs [116]. Thus, in the delivery of needling for sciatica and the mechanistic complications related to NP, an intersegmental approach is suggested (Figure 3).

### 4.5. Noradrenergic Modulation of NP

Noradrenaline (NA) has proved to alleviate NP symptoms resulting from sciatica nerve injury in rats [117]. Needle stimuli at Aδ threshold in the hindlimb of rats activates descending NA containing neurons from the locus coeruleus (LC), enhancing NA levels in the dorsal horn [118]. At the spinal level, NA inhibits nociceptive input from primary afferents and facilitates descending inhibition by activating α_1_ and α_2_ adrenoceptors, respectively [119,120]. NA reduces noxious inputs arriving at the dorsal horn by activating inhibitory or hyperpolarizing excitatory- interneurons and through inhibition of glutamate release from primary afferents [121,122]. In patients with sciatica, pain reduction following EA is correlated with increased arginine vasopressin levels (AVP) [123] as AVP triggers descending pain inhibition following increased NA levels in the hypothalamus [124,125]. Importantly, NA containing fibers from the LC of the hypothalamus project down to the dorsal horn to provide the primary supply of NA in the spinal cord [120]. NA may exert its presynaptic and postsynaptic effects by volume transmission in the dorsal horn, diffusing from its source and spreading to sites of action on afferent terminals and interneurons [126]. Theoretically, a hypothesis could be that needle stimulation to activate afferents along several rostro-caudal segments may potentiate analgesic effects as a result of NA volume transmission.

### 4.6. Restorative Effects of Needling for Neuropathic Pain

Injury of the sciatic nerve leads to activation of microglia and astrocytes in the lumbar dorsal horn [127,128]. At injured spinal levels, microglia accumulate and secrete brain-derived neurotrophic factor (BDNF) [129]. BDNF binds to the tyrosine kinase receptor B (TrkB) and when activated this pathway reduces KCC2 expression in DHN [130]. Consequently, the loss of KCC2 receptors limits CL- extrusion in DHN altering the inhibitory capacity of GABA and glycine [131,132]. Acute and prolonged release of BDNF initiates and sustains the CL- dysregulation that causes the symptoms characteristic of neuropathic pain [129]. Several studies support that following nerve injury, EA inhibits the expression of both microglia and astrocytes in the dorsal horn [127,128]. Tu et al. demonstrated EA reduced microglial activation and diminished BDNF and TrkB expression in the L4-L6 segments of the superficial dorsal horn in animal models of sciatic nerve injury [133]. In another study, EA down-regulated the BDNF and TrkB pathway following nerve injury while reducing the hyperexcitability of DHN [134]. Perhaps the most restorative effect of EA, is it substantially increased KCC2 expression of the lumbar dorsal horn, within the segments that received injured afferent fiber input [135]. Mechanistically, electric [136] and manual forms of needle stimuli [76] elicit distinct neurophysiological effects [137]. Yet, at the spinal nerve level both high and low frequency firing patterns, similar to those produced by EA, are also evoked by manual stimuli [80]. Previous studies have proved manual acupuncture to also inhibit microglia and astrocyte activation following spinal cord injury [138,139,140]. Taken together, studies support needling interventions to have therapeutic effects on patients with sciatica through their ability to inhibit spinal glial cells while restoring chloride regulation in DHN following sciatic nerve injury.

### 4.7. Limitations

Our scoping review has some limitations. First, the majority of articles that were included in two of the systematic reviews were written in Chinese or another language and full text could not be read. This limited our ability to further investigate treatment rationales and patient inclusion criteria. Second, several of the systematic reviews and meta-analysis studies included the same articles in the pooling of data for their results and limited our ability gain further information from several reviews. In contrast, this is the first scoping review associating the underlying mechanisms of NP with a clinical reasoning for the application of needling therapies in the management of sciatica.

## 5. Conclusions

This scoping review demonstrates that NP mechanisms are not routinely considered in needling approaches for patients with sciatica. The selection of needling locations and specific treatment parameters are currently based on TCM theory and expert consensus. Needles are typically inserted along the sciatic nerve distribution and associated dermatomes and/or myotomes. While studies show that acupuncture is an effective treatment option for sciatic pain, further research is warranted to explore if needling interventions for sciatica and NP would be more effective if NP mechanisms are considered. Further studies might consider that increasing concentrations of adenosine and NA through multi-segmental afferent input via needling, could be an effective strategy in patients with sciatica and NP. Importantly, needling interventions may reverse the disinhibition that occurs following nerve injury due to inhibition of spinal microglia and the restorative effects on chloride regulation.

## Figures and Tables

**Table 1:** Database formulas during literature search.

PubMed/MEDLINE Search Formula
(“dry needling” OR acupuncture) AND (sciatica OR “neuropathic pain” OR radiculopathy)
**CINAHL**
(“dry needling” OR “dry needling” OR acupuncture OR acupuncture) AND (sciatica OR “neuropathic pain” OR “neuropathic pain” OR radiculopathy)
**Cochrane Database of Systematic Reviews**
**# 1** acupuncture OR “dry needling” OR (mh acupuncture)
**# 2** sciatica OR “neuropathic pain” OR radiculopathy (MeSh)
**# 3** # 1 AND # 2

**Table 2:** Inclusion and exclusion criteria.

Scoping Review Studies
**Inclusion criteria**
**Language:** English
**Article type:** Systematic reviews, meta-analyses, randomized controlled trials, pilot studies
**Subject:** Dry needling or acupuncture for sciatica or neuropathic pain related to radiculopathy of lumbar region due to sciatic nerve compression
**Exclusion criteria**
**Language:** Non-English language
**Article type:** Case series, case study, cohort study, study protocol, narrative review, articles not in English
**Subject:** Mechanisms studies or experimental animal studies, studies on low back pain or spinal pain without specifying patients with nerve root compression and symptoms consistent with sciatica. Studies on patients with lumbar spinal stenosis. Studies on patients with neuropathic pain not of spinal or nerve root origin to include; chemotherapy-induced peripheral neuropathy, spinal cord injury, multiple sclerosis, cancer related neuropathic pain, post herpetic neuralgia, piriformis syndrome, diabetic neuropathy. Studies using warming acupuncture or injectates with the needling procedures
**Mechanisms Studies**
**Inclusion Criteria**
**Language:** English
**Article type:** Systematic reviews, meta-analyses, randomized controlled trials or pilot studies, narrative reviews, experimental in vivo animal studies, clinical studies, laboratory studies, modeling and simulation studies.
**Subject:** Physiological mechanisms studies (human and animal subjects) on the use of dry needling, manual acupuncture or electroacupuncture for neuropathic pain related to sciatic nerve injury/compression models. Mechanistic studies on animal models under normal, inflammatory and/or neuropathic pain conditions
**Exclusion criteria**
**Language:** Non-English language
**Article type:** Studies not relevant to the neurophysiological or mechanical effects of needling interventions. Studies not relevant to pathophysiological mechanisms of sciatica, nerve injury or neuropathic pain.

**Table 3:** Description of included studies.

Study	Study Design	Number of Patients	Pain Outcome	Follow Up	Rationale	Inclusion Criteria
Ji et al., 2015 [44]	Systematic Review and Meta-Analysis.	12 Studies (randomized or quasi-randomized clinical trials) involving 1842 participants	VAS (*n* = 3)	Not reported	All 12 studies used TCM rationale for point selection	Studies chose participants with either subjective signs of sciatica or positive clinical examination tests or both. Conformity was limited on inclusion criteria among studies.
Huang et al., 2020 [40]	Systematic Review and Meta-Analysis.	24 RCTs included in systematic review, 22 RCTs in Meta-analysis. Only 3 RCTs on sciatica involving 196 patients	VAS (*n* = 2), NRS (*n* = 1)	Kim et al., 2016: weeks 6 and 12, see below for Huang et al. [41] and Zhang et al. [38]	Kim et al., 2016: point selection was at the discretion of Korean Medical Doctors and was individualize. See below for Huang et al., 2019 and Zhang et al., 2017	Kim et al., 2016: required clinical and radiological confirmation along with symptoms of radiating pain in the leg. See below for Huang et al. [41] and Zhang et al. [38]
Huang et al., 2019 [41]	RCT	44 patients	VAS	Weeks 1, 2, 3, 4, 16, and 28. Primary outcome was VAS at 4 weeks.	Selection of points was based on expert consensus and protocol of a previous trial.	Patients with chronic sciatica caused by lumbar disc herniation. Diagnosis was based on MRI, CT and examination of symptoms by experienced physicians.
Lewis et al., 2015 [24]	Systematic Review and Network Meta-Analysis. 122 studies included	Only a single RCT on acupuncture was included, Duplan, 1983 (French) involving 30 patients	No data reported.	No data reported	Not reported	Patients with clinical diagnosis of sciatica based on nerve root pain and referred pain
Liu et al., 2019 [22]	Randomized Controlled Pilot Study	30 patients	VAS	4 weeks	Acupoint selection was based on acupuncturist experience and TCM theory. However, sciatic dermatomes were considered in point selection	Patients selected based on radicular pain in L4, L5, S1 dermatomes, findings of radicular pain, motor, sensory or reflex deficits on neurological exam, positive SLR, leg pain upon sneezing, coughing or straining and positive MRI showing unilateral disc herniation with impingement on L4, L5 or S1 nerve root.
Luijsterburg et al., 2007 [43]	Systematic Review. 30 publications included	Only a single RCT on acupuncture was included, Duplan, 1983 (French) involving 30 patients	No data reported.	No data reported	Not reported	Patients with clinical diagnosis of sciatica based on nerve root pain and referred pain
Qin et al., 2015 [45]	Systematic Review and Meta-Analysis	11 RCTs included with 932 participants. 9 were in Chinese, 2 were in English	VAS (*n* = 3)	Reported only in 1 study as 6 months	All studies adopted a treatment theory based on TCM theory and clinical experience.	Patients with sciatica of the nerve roots along with lumbar disc herniation (*n* = 8 studies). Patients diagnosed with sciatica of the nerve trunk without lumbar disc herniation (*n* = 3 studies)
Zhang et al., 2017 [38]	RCT	100 patients	NRS	Weeks 1, 2, 3, 4, 16, and 28. Primary outcome was meanchange in NRS at week 4	Protocol based on specialist consensus and results of a previous pilot trial	Included participants with sciatica symptoms that correlated with MRI or CT findings of lumbar disc herniation
Jeong et al., 2020 [39]	RCT	146 patients	VAS	Weeks 2, 4 and 6. Primary outcome was mean change in VAS at week 4	Acupuncture rationale not specified	Included patients diagnosed with LDH based on clinical examination with positive MRI or CT and symptoms of low back pain, radiating pain, and paresthesia or weakness in the lower extremities
Lewis et al., 2011 [42]	Systematic Review. Cost-effectiveness of treatments for sciatica. 270 studies	Only a single RCT on acupuncture was included, Duplan, 1983 (French) involving 30 patients	No data provided	No data reported	Not reported	Patients with clinical diagnosis of sciatica based on nerve root pain and referred pain

VAS: visual analog scale 0–100 mm; NRS: 11-point numeric rating scale; TCM: traditional Chinese medicine; RCT: randomized controlled trial; MRI: magnetic resonance imaging; CT: Computed tomography; LDH: lumbar disc herniation.

**Table 4:** Description of needling interventions.

Study	Interventions	Needle Placement	Needle Manipulation	Retention Time	Frequency/ Duration
Ji et al., 2015 [44]	MA or EA vs. Conventional Western Medicine (oral drugs, external drugs or injections)	Common points: GB 30 (*n* = 12 studies), BL 54 (*n* = 7 studies), BL 40 (*n* = 8 studies), GB 34 (*n* = 5 studies), BL 25 (*n* = 6 studies), BL 23 (*n* = 5 studies), BL 60 (*n* = 8 studies), BL 57 (*n* = 6 studies), GB 39 (*n* = 6 studies)	Manual stimulation (*n* = 8 studies) electric stimulation (*n* = 4 studies) 10 out of 12 studies elicited de qi or other sensation	Ranged from 5 to 30 min for either MA or EA	Number of sessions ranged from 6 to 40. Frequency ranged from once per day ×6–15 days to 2 times per week for 3 weeks to 3 times per week for 2 weeks
Huang et al., 2020 [40]	MA vs. Sham Acupuncture, EA vs. Medium Frequency Electrotherapy (MFE), MA + EA vs. usual care alone (Physical Therapy)	Huang et al., 2019 [41]: (B) BL 23, BL 25, BL 40, BL 57). Zhang et al. [38]: BL 25 on affected side, Jiaji (Ex-B2) bilaterally at spinal level of lumbar disc herniation. Kim et al., 2016: BL23, BL24, BL25 or BL26 or Jiaji points at L2–L5 spinal levels. Other used points were BL57, BL60, GB39, GB34 and tender points	Kim et al., 2016 Manual stimulation 15–50 mm depth, lift-thrust and needle rotation to elicit de qi. Electrical stimulation applied with alternating 2–100 Hz frequency	Kim et al., 2016 retention time 20 min with EA 2–100 Hz alternating. See below for Huang et al. [41] and Zhang et al. [38]	Kim et al., 2016 = 12–16 sessions over a 6-week period. See below for Huang et al. [41] and Zhang et al. [38]
Huang et al., 2019 [41]	MA (*n* = 23) vs. Sham Acupuncture (*n* = 21)	Acupuncture to (B) BL 23, BL 25, BL 40, BL 57. Sham group used blunt needles on same points without insertion	Manual stimulation, depth of needling 40–70 mm into BL 25, 30 mm into BL 40 and BL 57 Needle twirling, lifting and thrusting were used to elicit de qi	30 min	3 ×/week for 4 weeks 12 sessions
Lewis et al., 2015 [24]	EA vs. sham acupuncture	No data reported	EA	Not reported	5 session of EA
Liu et al., 2019 [22]	High dose MA vs. Low dose MA	High Dose = 18 points BL 23, BL 25, BL 27, GB 30, BL 37, BL 54, BL 36, GB 31, BL 40, ST 36, GB 34, SP 9, BL 58, SP 6, GB 39, BL 60, KI 3, BL 62. Low Dose = 6 points BL 23, GB 30, BL 40 GB 34, BL 60, GB 39	Manual stimulation = needle rotation at 5–30 mm depth and elicited de qi	20–30 min	2 ×/week for 4 weeks 8 sessions
Luijsterburg et al., 2007 [43]	30 patients with sciatica (15 in acupuncture group and 15 placebo acupuncture)	No data reported.	EA	Not reported	5 session of EA
Qin et al., 2015 [45]	MA (*n* = 2 studies), EA (*n* = 6) studies, Warming Acupuncture (*n* = 2 studies). Comparison interventions included; conventional medication (*n* = 8 studies), acupuncture with meds compared to meds alone (*n* = 2 studies), 1 trial compared acupuncture with sham acupuncture	Number of points used ranged from 1 to 10 across studies. Most commonly used points were GB 30 (*n*= 9 studies) BL 40 (*n* = 8 studies), BL 67 (*n* = 4 studies). Other common points were BL 54 (*n* = 4 studies), Jiaji (EX-B2) (*n* = 6 studies), BL 57 (*n* = 3 studies), BL 23 + BL 25 (*n* = 2 studies)	MA (*n* = 2 studies). EA (*n* = 6 studies). All 11 studies reported de qi needle sensation of soreness and numbness	Retention time varied from 20–45 min	1 to 4 weeks. Frequency ranged from 1 to 3 sessions per day for 7–10 days (*n* = 9 studies) or 2 to 4 sessions 3 times per week (*n* = 2 studies)
Zhang et al., 2017 [38]	EA (*n* = 50) vs. MFE (*n* = 50)	BL 25 on affected side, Jiaji (Ex-B2) bilaterally at spinal level of lumbar disc herniation. MFE = surface electrodes applied over same points as acupuncture group	Manual stimulation (BL 25 up to 3 inch depth and Jiaji (Ex-B2) up to 1.5 inch depth, + electrical stimulation = 50 Hz	20 min	5 times per week for 2 weeks then 3 sessions per week for 2 weeks.
Jeong et al., 2020 [39]	MA (*n* = 73) vs. Acupotomy (*n* = 73)	MA = GV 3 and (B) BL 23, BL 24, BL 25, BL 26, GB 30, BL 40, BL 60 Acupotomy = 2–6 points at lumbar levels of disc herniation	MA = Manual needle rotation 3–5 times after insertion 20 mm for BL 40 and BL 60, 30 mm depth for all others. Acupotomy = 50–70 mm depth to 2–6 points	MA = 15 min Acupotomy = immediate removal after manipulation	MA = 4 sessions over a 2-week period
Lewis et al., 2011 [42]	30 patients with sciatica (15 in acupuncture group and 15 placebo acupuncture).	No data provided	EA	No data provided	5 session of acupuncture

MA: manual acupuncture; EA: electroacupuncture; BL: bladder; Jiaji: huatuojiaji; GB: gallbladder; Hz: hertz; ST: stomach; SP: spleen; KI: kidney.
